# Nanoparticles in the treatment of osteoporosis: recent advances in nanoparticles for the treatment of osteoporosis

**DOI:** 10.1039/d5ra06586k

**Published:** 2025-10-28

**Authors:** Yingying Li, Zihui Liu, Wenqing Wang, Junjie Li, Jieping Zhao, Cory J. Xian, Yuankun Zhai

**Affiliations:** a Henan Luoyang Orthopedic Hospital (Henan Provincial Orthopedic Hospital) Luoyang 471000 China yylykz@163.com; b School of Stomatology, Henan University Kaifeng 475001 China; c Kaifeng Key Laboratory of Periodontal Tissue Engineering Kaifeng 475000 China; d UniSA Clinical and Health Sciences, University of South Australia Adelaide SA 5001 Australia

## Abstract

Osteoporosis (OP) is a systemic skeletal disease characterized by reduced bone mass, deterioration of bone microarchitecture, and a significantly elevated risk of fractures. With the intensification of population aging, the incidence of OP continues to rise, making it a major global public health concern. Traditional or conventional therapeutic regimens for OP primarily rely on calcium and vitamin D supplementation combined with bisphosphonates or hormone replacement therapy, which have some obvious limitations including side effects. Oral bisphosphonates tend to irritate the oesophageal mucosa, and long-term use of hormone drugs may increase the risk of cardiovascular disease and cancer. In recent years, nanoparticles (NPs) have become an emerging direction in the treatment of OP due to their unique physicochemical properties and biocompatibility. This article has systematically reviewed the research progress of NPs in this field. First, we summarized their fundamental characteristics, Furthermore, this article reviewed various types of NPs applied in OP management, and analyzed their respective advantages, limitations, and clinical application prospects. Ultimately, this study not only summarizes the current landscape but also charts a future course by proposing developmental pathways and clinical translation strategies. This review aims to provide a clear theoretical basis and directional guidance for future researchers in designing efficient and safe nanotherapeutic systems, thereby accelerating the clinical application of nanotechnology in the field of precision treatment for OP.

## Abstract 2

Osteoporosis (OP) is a systemic skeletal disease characterized by reduced bone mass, deterioration of bone microarchitecture, and a significantly elevated risk of fractures.

## Graphical abstract

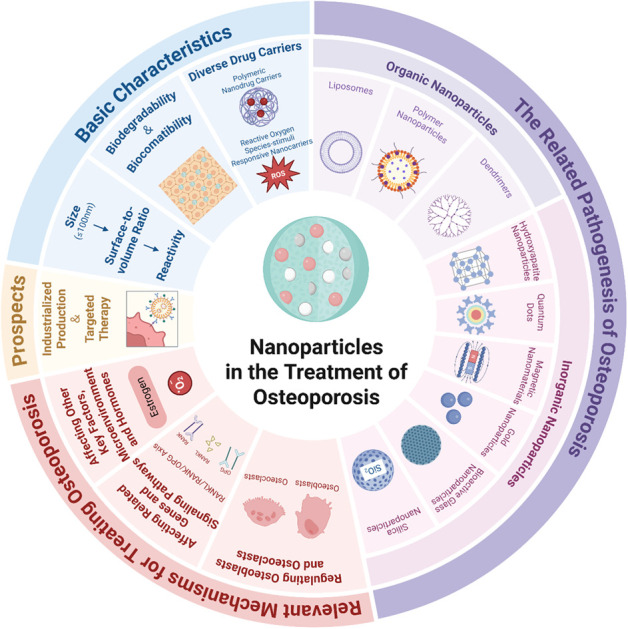

## 1. Introduction

Osteoporosis (OP) is a systemic skeletal disease characterized by reduced bone mass, degeneration of bone microstructure, and a significantly increased risk of fractures.^1^ The pathogenesis is the result of a complex interplay among genetic, epigenetic, endocrine, environmental, and lifestyle factors.^2^ Statistics show that approximately one-third of individuals aged 60 to 70 suffer from OP, and more than half of those over 80 years old are affected by the condition.^3^ The incidence of OP is increasing with the growing challenge of an aging population, making it a significant global public health concern.

Traditional treatment for OP is based on calcium (Ca) and vitamin D supplementation, often in conjunction with bisphosphonate (Bis) or hormone replacement therapy. However, these approaches have obvious limitations. For instance, oral Bis exhibits low bioavailability and can irritate the esophageal lining due to its acidic nature, requiring patients to remain upright for 30 to 40 minutes after ingestion. This requirement can be especially challenging for elderly patients with mobility issues. Individuals with renal insufficiency (creatinine clearance rate <35 mL min^−1^) should also exercise caution when taking oral Bis.^4,5^ Furthermore, long-term use of hormonal medications may increase the risk of cardiovascular disease and cancer.^6^ Similarly, biopharmaceuticals such as denosumab (a RANKL inhibitor) have been developed for OP therapy, yet concerns regarding their long-term efficacy and safety persist.^7^

In recent years, a variety of novel therapeutic strategies have been gaining increasing attention. As platforms for drug and ion delivery, metal–organic frameworks (MOFs) utilize metal ions such as zinc, strontium, and magnesium to achieve local osteogenesis and inhibit osteoclastogenesis.^8^ However, their structural stability and the potential toxicity of degradation products remain key barriers to clinical translation into therapies for OP. Injectable hydrogels, through minimally invasive injection, enable the sustained release of drugs, factors, and stem cells, significantly enhancing the efficacy of bone defect repair.^9^ However, their insufficient mechanical strength and the biosafety of the gelation process still require careful consideration. In terms of gene therapy, approaches based on delivering microRNAs to cells have emerged as effective treatments for various diseases.^10–12^ In OP, a variety of miRNAs have been demonstrated to play a crucial role by regulating the key signaling pathways involved in the differentiation of bone-forming cells osteoblasts and bone resorptive cells osteoclasts, highlighting their therapeutic potentials as core nodes in the regulatory networks of bone metabolism. However, the application of miRNAs is unfortunately constrained by difficulties in their cellular uptake and poor stability.^13^

Against this backdrop, nanomedicine, as an emerging interdisciplinary field, has demonstrated significant application prospects nanoparticles (NPs) not only possess the capabilities for precise targeting and efficient drug delivery, but they also overcome the stability and cellular endocytosis limitations of conventional miRNA therapy, offering new therapeutic strategies for diseases such as OP. Studies have shown that NPs, as drug carriers, have achieved preliminary success in both passive and active targeting of tumors and cancer cells.^14^ They enable controlled drug release through synergistic action of multiple mechanisms, including diffusion, degradation, swelling, and stimulus-responsiveness. This significantly enhances their targeting specificity and loading efficiency, establishing them as core components of intelligent drug delivery systems. Furthermore, the physicochemical properties of NPs critically influence their biodistribution, cellular uptake, and pharmacokinetics.^15^ By encapsulating proteins, systems like liposomes, polymeric NPs, and gold NPs achieve controlled drug release and targeted delivery, thereby overcoming challenges such as the poor bio-stability and low delivery efficiency of protein therapies.^16^ In bone tissue engineering, NPs mimic the nanostructure of the natural bone matrix, providing a suitable microenvironment for bone cell growth.^17^ They also significantly enhance the function of bone repair scaffolds by improving cell adhesion, promoting osteogenic differentiation, stimulating angiogenesis, and improving biocompatibility.^18^ These studies have laid a solid theoretical foundation for the use of NPs in the intervention of OP, demonstrating their key role in advancing bone tissue engineering. This article aims to summarize the fundamental properties of NPs and their applications in OP. Additionally, it reviews their mechanisms of action and discusses their potential and prospects for future practical use.

## 2. Basic properties

### 2.1 Size and surface properties of NPs

The dimensions and surface characteristics of NPs play a crucial role in their applications. Although the 2011/696/EU directive has relaxed the traditional size determination threshold for nanomaterials, it still defines them as substances with an external size not exceeding 100 nm in at least one dimension. It is noteworthy that in mixed particle systems, as long as nanoscale particles (≤100 nm) meet the criteria in terms of their numerical proportion, the entire system can still be classified as a nanomaterial.^19^

NPs exhibit unique physical and chemical properties due to their diminutive sizes, distinguishing them from larger bulk materials. NPs have a high surface-to-volume ratio, which significantly enhances their reactivity and interaction with biological systems. Modifications of the surface chemistry of NPs can alter their solubility, stability, and targeting ability. This surface modification property is particularly crucial in the field of drug delivery, where the surface characteristics of NPs can determine the pharmacokinetics and biodistribution of therapeutic agents.^20^ Furthermore, the shape and size of NPs can affect their cellular uptake^21^ and tissue penetration, which are key factors influencing the efficacy of NPs. Recent studies indicate that NPs with optimal size and surface characteristics can facilitate targeted delivery to specific tissues, such as bone tissue, thereby enhancing the therapeutic effect while minimizing side effects.^22,23^ Currently, the ability to precisely control the surface engineering of NPs has become attainable, which is essential for advancing the development of nanomedicine approaches and will drive future innovations in the field.

### 2.2 Biocompatibility and biodegradability

Biocompatibility and biodegradability are significant characteristics of NPs. The biocompatibility of these NPs is primarily influenced by systemic responses to drug administration and the toxic effects of the NPs and their metabolites on various organs. Meanwhile, biodegradability is determined by the traits of the NPs, the duration of their presence in the body, and the metabolic processes involved. Researchers have utilized these characteristics to develop numerous new drugs for the treatment of diseases, addressing the toxic side effects caused by the low biocompatibility and low biodegradability of traditional medications. For example, prolonged use of Bis, a traditional medication for treating OP, can result in adverse effects such as osteonecrosis of the jaw due to the accumulation of the drug in the bone.^24^ Researchers utilized poly(lactic-*co*-glycolic acid) (PLGA) NPs as carriers for drug delivery, incorporating Bis into these NPs, so that the exceptional biocompatibility and biodegradability of PLGA NPs were capitalized to reduce the toxic effects of Bis on the body caused by their accumulation.^25,26^

### 2.3 Diversity of drug carriers

**Fig. 1:**
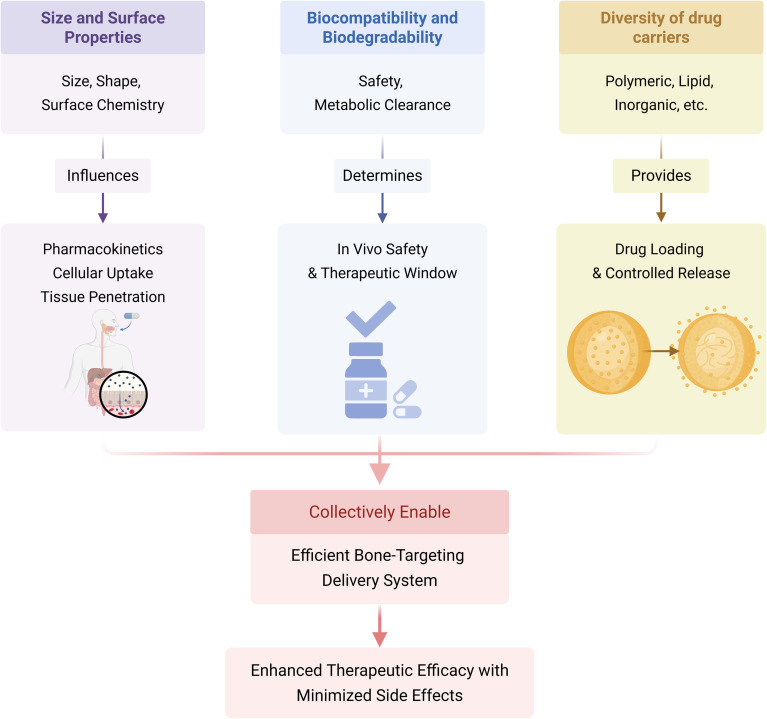
Fundamental properties of nanoparticles. The fundamental properties of nanoparticles include size and surface engineering, biocompatibility and degradability, as well as carrier diversity. By influencing their downstream functions, these properties jointly determine their final efficacy in osteoporosis treatment. Created in https://BioRender.com.

With the continuous advancement of nanotechnology, the development and application of nano-drug carriers in the medical field are continually expanding.^27^ It is this diversity of nano-drug carriers that offers an essential basis for precision drug delivery. Currently, the main types of nano-drug carriers include polymeric nano-carriers,^28^ reactive oxygen species (ROS)-stimulated responsive nanocarriers,^29^ non-viral nano-drug carriers,^30^ and thermosensitive nano-drug carriers, among others. These nano-drug carriers offer several advantages, including enhanced drug targeting, improved drug stability and solubility, and a reduction in drug toxicity and side effects. They can meet the delivery requirements of various medications and have shown significant promise in areas such as cancer treatment,^31^ atherosclerosis intervention,^32,33^ and brain disease management.^34,35^ Their potential is considerable, offering innovative approaches for disease treatment and a broad range of applications in the medical field^27,36^ (Fig. 1).

## 3. Different types of NPs in treating OP

In the research on osteoporosis (OP) treatment, traditional drug delivery is hindered by issues such as weak bone-targeting, low bioavailability, and non-target organ toxicity, making it difficult to achieve the goal of “acting precisely on bone lesions”. However, nanodrug delivery systems, with their controllable microstructure, efficient drug-loading capacity, and customizable functional modification, provide key support for breaking through this dilemma and have become one of the core research directions in current OP treatment. Classified by core material and chemical composition, NPs used for OP treatment are mainly divided into two categories: organic and inorganic. The following text will elaborate on their specific types, core properties, and applications in OP treatment in detail (Table 1).

**Table 1:** Key characteristics and application correlations of nanoparticles in osteoporosis treatment

Categories of nanoparticles	Specific types	Core structure/properties	Main loaded drugs/active ingredients	Mechanism of action related to osteoporosis treatment	Key therapeutic effects	Reference
Organic NPs	Liposomes	Phospholipid bilayer vesicles with hydrophilic ends facing outward and hydrophobic ends forming the inner core; stability and targeting can be regulated by cholesterol/polymers, capable of encapsulating hydrophilic/hydrophobic/amphiphilic drugs	Linagliptin, teriparatide, PA, puerarin, IQ, specific miRNAs, zinc finger transcription factor ZEB1	(1) Bone-targeted drug delivery; (2) delayed drug release and maintenance of drug stability; (3) activation of Wnt/β-catenin, Notch and other signaling pathways; (4) regulation of osteoclast formation and bone resorption	(1) Improvement of bone structure parameters and BMD; (2) inhibition of osteoclast activity and promotion of bone anabolism; (3) reduction of systemic cytotoxicity; (4) achievement of synergistic gene therapy	37–53
	Polymeric NPs	Nanocapsules: the drug core (aqueous/oil phase) is encapsulated by a polyester film, and some drugs are attached to the surface; nanospheres: drugs are uniformly dispersed in a polymer matrix (such as PLGA) to form an integrated drug-loaded structure; both have high surface activity and customizable physical and chemical properties	SPIO:Eu, siSema4D (targeting the Sema4D gene), RANK siRNA, PEI	(1) Nanospheres: magnetic field-guided bone targeting, regulation of macrophage polarization to restore bone metabolic balance; (2) nanocapsules: silencing the Sema4D gene to block the signaling pathway and down-regulating Th17 differentiation and pro-inflammatory factors; (3) controlled drug release and reduction of systemic toxicity	(1) Repair of trabecular microstructural damage; (2) dual regulation of bone metabolism and immune homeostasis; (3) inhibition of osteoclast differentiation and activity; (4) optimization of drug controlled-release performance; (5) reducing systemic toxicity and improve bone-targeting efficiency	54–62
	Dendrimers	Nanoscale in size, with a hierarchical branched structure forming internal cavities; surface functionalization is controllable, excellent biocompatibility, and can efficiently encapsulate hydrophobic drugs	Cur, polyethylene glycol-conjugated phosphorylated serine-modified poly(amidoamine)	(1) Targeting the surface of mouse bone tissue (osteoid-targeted); (2) pH-responsive drug release (dissociation in the acidic environment of lysosomes); (3) improvement of the solubility and bioavailability of hydrophobic drugs	(1) Solving the problems of poor water solubility and high toxicity of curcumin; (2) inhibition of osteoclast formation and promotion of bone formation; (3) achievement of precise drug delivery	63–71
Inorganic NPs	HA NPs	Highly similar to the mineral in the bone matrix, capable of forming chemical bonds with bone tissue; excellent biocompatibility and bone-targeting; performance can be optimized by ion doping/structural modification	Salmon calcitonin (SCT), zoledronic acid, PTH (1–34), zinc ions	(1) Direct binding of anti-bone resorption drugs for bone-targeted delivery; (2) ion doping (such as Zn^2+^) to regulate bone metabolism; (3) drug release by dissolution at the optimal pH (6.8) for osteoclast activity	(1) Improvement of mucosal permeability and drug accumulation in bone tissue; (2) enhancement of the mechanical strength of the femoral shaft and maintenance of the microstructures of the bone cortex/trabeculae; (3) restoration of bone metabolic balance and increase of BMD	72–82
	Quantum dots	Nanoscale inorganic semiconductor particles with unique fluorescence properties; can be compounded with biological materials to construct functional coatings	Copper oxide quantum dots (CuO QDs)/chitosan (CS), graphene oxide quantum dots (GOQD)/layered double hydroxide (LDH)	(1) Interaction with the active ingredients of *Angelica sinensis* to enhance the activity of osteoblasts; (2) activation of mitophagy to clear abnormal mitochondria; (3) improvement of the local microenvironment for osteogenesis	(1) Inhibition of osteoblast apoptosis; (2) enhancement of bone regeneration and osseointegration effects; (3) provision of an OP bone defect treatment plan for magnesium alloy implants	83–85
	Magnetic NPs	Containing Fe/Ni/Co and their metal oxides, with a high specific surface area and controllable magnetism; can be used as MRI contrast agents and drug carriers	Iron oxide nanoparticles (IONPs), Bis/dextran/Fe_3_O_4_, bone marrow mesenchymal stem cell exosomes (loaded with miR-150-5p)	(1) Regulation of the TRAF6-Sequestosome 1-Cylindromatosis signaling complex to inhibit osteoclasts; (2) radiofrequency heating to induce osteoclast lysis; (3) magnetically targeted delivery of bioactive molecules	(1) Achievement of integrated diagnosis and treatment of OP (MRI contrast + treatment); (2) inhibition of osteoclast survival and bone resorption; (3) promotion of osteoblast proliferation and maturation (especially in diabetic OP)	86–97
	Gold NPs	The most stable metal NPs, with good biocompatibility and low toxicity; can be modified with functional groups or compounded with polymers	Alendronate sodium, curcumin (Cur), α-lipoic acid, SOST siRNA, silver (Ag), pleiotrophin	(1) Inhibition of RANKL/M-CSF receptors to reduce osteoclast activity; (2) clearance of excessive intracellular ROS and up-regulation of osteogenic genes (BMP-2, Runx2); (3) gene silencing (down-regulation of SOST)	(1) Maintenance of bone microstructure and strength and prevention of bone resorption; (2) promotion of bone regeneration and fracture healing; (3) safe implementation of OP gene therapy; (4) inhibition of osteoclast formation without cytotoxicity	98–110
	Bioactive glass NPs	Silicate-based amorphous materials, mesoporous structures (MBG NPs) have a high specific surface area; can release Ca^2+^/Si^4+^ to promote bone regeneration	Strontium (Sr), icariin, estradiol (E2), boron (B)	(1) Continuous release of ions (Sr^2+^/Si^4+^) to regulate bone metabolism; (2) sustained release of drugs (such as estradiol) through the mesoporous structure; (3) maintenance of the integrity and bioactivity of the composite scaffold	(1) Dual functions: promotion of osteoblast proliferation and inhibition of osteoclasts; (2) repair of irregular bone defects; (3) reduction of bone resorption-related markers (such as tartrate-resistant acid phosphatase)	111–118
	Silica NPs	Divided into mesoporous (MSNs) and core/shell types, with excellent chemical stability and good biocompatibility; the mesoporous structure has a high drug-loading capacity	Pleiotrophin, mercapto (–SH), calcium (Ca^2+^), gallium (Ga^3+^)	(1) Mercaptolation to neutralize ROS and reduce cell damage; (2) surface modification with PEI to enhance cell viability and osteogenic gene expression; (3) Ga doping to inhibit macrophage differentiation into osteoclasts	(1) Induction of bone formation without the need for osteogenic supplements; (2) breaking through the limitations of traditional osteogenic culture media; (3) enhancement of osteogenic activity	119–125

### 3.1 Organic NPs

#### 3.1.1 Liposomes

Liposomes are bilayer vesicle structures formed spontaneously by phospholipid molecules in an aqueous environment through hydrophobic interactions. The fundamental structure consists of a phospholipid bilayer, with hydrophilic heads oriented toward the external aqueous environment and hydrophobic tails converging to form an inner core region.^37^ This oriented arrangement of the structure not only stabilizes the morphology of the vesicles but also confers the capability to encapsulate hydrophilic, hydrophobic, and amphiphilic drugs. By incorporating cholesterol or polymers, the stability and targeting capability of liposome membranes can be finely tuned, thereby broadening the scope of drug delivery applications. This liposome delivery approach not only markedly improves the therapeutic outcomes of drugs but also minimizes their adverse effects.^38,39^

In the treatment of diabetes-related OP, bone-targeted liposomes modified with aspartic acid conjugates poly(aspartic acid-*co*-lactide)-1,2-dipalmitoyl-*sn*-glycero-3-phospho ethanolamine effectively enabled the targeted delivery of linagliptin. This system improved bone structural parameters and bone mineral density (BMD) by enhancing linagliptin's bone-binding capacity, delaying drug release, and maintaining pharmaceutical stability.^40^ Furthermore, thermo-responsive liposomes constructed with temperature-sensitive phospholipids achieved the controllable release of teriparatide through local temperature changes, thereby enhancing its promotion of bone anabolism.^41^

This liposome delivery approach has been successfully used to improve the application in treating OP of some monomer compounds derived from traditional Chinese medicine. While the monomer pomolic acid (PA) derived from the medicinal herb *Salvia divinorum* can treat OP by inhibiting osteoclastogenesis, the lack of its targeting ability leads to a weakening of the therapeutic effect.^42^ Researchers developed a novel bone-targeting nanomedicine, alendronate-functionalized polyethylene glycol liposomes, by encapsulating PA within alendronate-functionalized liposomes. This system significantly enhances the bone-targeting efficiency of PA. While this system inhibited osteoclastogenesis and bone resorption, it successfully reduced systemic cytotoxicity. Its anti-bone loss effect was also verified in the mouse model of OP.^43^ Similarly, puerarin, one of the main components of *Pueraria lobata*, can inhibit osteoclastogenesis by suppressing the Tumor Necrosis Factor Receptor-Associated Factor 6 (TRAF6)/ROS-dependent mitogen-activated protein kinases (MAPK)/Nuclear Factor kappa-light-chain-enhancer of activated B cells (NF-κB) signaling pathway^44,45^ and promote osteogenesis by enhancing osteogenesis-related signaling pathways such as Wnt/β-catenin.^46^ Researchers employed liposome encapsulation and targeted modification with d-α-tocopherol polyethylene glycol 1000 succinate (TPGS) to prepare long-circulating liposomes of puerarin (TPGS-puerarin-liposome). In rats, this composite not only enhanced the blood concentration of puerarin through sustained release but also activated the Wnt/β-catenin signaling pathway. Finally, isoquercetin (IQ) is a widely occurring natural phytoestrogen that can be utilized for the treatment of OP.^47–49^ Polyethylene glycolated long-circulating liposomes containing IQ exhibited significant anti-osteoporotic potential in a de-ovulated rat model by enhancing bone microstructural density and resistance to oxidative stress.^50^

The liposome delivery approach has also found utilization in the field of gene therapy of OP. The alendronate gene–lipid complex, which is composed of a alendronate-functionalized liposomal carrier and specific miRNAs, was found to control osteoclastogenesis and bone resorption.^51^ Similarly, liposomes encapsulating the zinc finger transcription factor ZEB1 effectively promoted angiogenesis-dependent osteogenesis and reduced bone loss by restoring the activity of the Notch signaling pathway in the bone endothelium.^52^ In addition, bone-targeting liposomal carriers have also been used to deliver miR-29b, which demonstrated a dual effect of promoting bone formation and inhibiting bone resorption in OP models.^53^

Therefore, liposomes, boasting their customizable nanostructures and other capabilities, have emerged as highly promising nano-drug carriers for the treatment of OP.

#### 3.1.2 Polymeric NPs

Polymeric NPs (PNPs) have demonstrated tremendous potential in the treatment of OP in recent years, owing to their unique physical properties and good biodegradability. PNPs are primarily classified into two main types, nanocapsules and nanospheres, based on their respective methods of drug binding. Nanocapsules consist of a polyester film that encapsulates a drug core. This polyester film seals the drug molecules within an inner aqueous or oily core, and a portion of the drug may also attach to the surface.^54^ In contrast, nanospheres disperse the drug uniformly within a polymer matrix, thereby constructing an integrated loading structure.^55,56^ With the advantages of having a high surface activity and highly customizable physicochemical properties, these two types of systems excel in optimizing drug-controlled release performance, enhancing bone tissue targeting efficiency, and reducing systemic toxicity. They provide innovative solutions for improving the therapeutic effect of OP.^57,58^

Nanosphere systems achieve drug overall loading by uniformly dispersing the drug within a polymer matrix. A typical example is the SPIO: europium (Eu)@PLGA composite nanosphere, in which Eu ions-doped superparamagnetic iron oxide (SPIO) nanocrystals are encapsulated within PLGA. Under the guidance of a magnetic field, this system can achieve bone targeting, enhance osteoblast differentiation, and inhibit osteoclastogenesis. More importantly, SPIO:Eu@PLGA nanospheres can effectively repair bone trabecular microstructural damage, which was achieved by modulating macrophage polarization and restoring the dynamic balance between bone resorption and bone formation.^59^ This magnetic-responsive therapy approach has thus opened new avenues for OP intervention.

Nanocapsule systems, by contrast, achieve the coordination of multiple functions *via* their core–shell structure. For example, the core–shell nanocapsule aALN-mLFN, composed of a cationized lactoferrin core and an alendronate polymer shell, precisely delivers small interfering RNA targeting the Semaphorin 4D gene (siSema4D) to bone tissue. By silencing the Sema4D gene, it blocks the Sema4D-Plexin-B1 signaling pathway, thereby inhibiting osteoclast differentiation and bone resorption.^60^ Simultaneously, it down-regulates the differentiation of Th17 cells and the release of pro-inflammatory factors such as Interleukin (IL)-17 and Tumor Necrosis Factor alpha (TNF-α). This approach synergistically enhances the bone microenvironment and immune regulation, achieving dual regulation of bone metabolism and immune homeostasis. Such a strategy can thus provide a highly effective and synergistic treatment for OP.^61^ Also, PLGA can be utilized to fabricate a siRNA nanocapsule delivery system by encapsulating polyethyleneimine (PEI)/Receptor Activator of Nuclear Factor κ B (RANK) siRNA complexes. This system downregulates the RANK mRNA expression level in osteoclast precursors and inhibits the differentiation and activity of osteoclasts.^62^

Thus, nanocapsules and nanospheres are unique in their structural designs, and both achieve controlled-release delivery and targeted delivery of drugs through their high surface activities. These two types of PNPs have significant advantages in reducing systemic toxicity and enhancing bone-targeting efficiency, which have opened up a new direction for the precision treatment of OP.

#### 3.1.3 Dendrimers

Dendrimers, with their nanoscale sizes,^63^ precisely controllable surface functionalization, and excellent biocompatibility, have emerged as ideal drug carriers for the treatment of OP.^64–66^ These materials can form internal cavities *via* hierarchical branching structures, which enable them to efficiently encapsulate hydrophobic drugs and thus achieve precise delivery and controlled release of pharmaceuticals. Taking the polyethylene glycol-conjugated phosphorylated serine-modified poly(amidoamine) dendrimer as an example, studies have verified its specificity for enrichment on the surface of mouse skeletal tissue. This characteristic positions it as a drug carrier targeting the surface of the osteoid.^67^ The precise bone tissue targeting ability of this dendrimer may provide new ideas for the treatment of OP and other related bone diseases.

Due to its dual regulatory effects of promoting bone formation and inhibiting bone resorption, curcumin (Cur), a natural compound, has emerged as a potential drug for the treatment of OP.^68,69^ However, its poor water solubility, low bioavailability, and significant toxicity to other organs largely limit their clinical translation.^70^ To address this issue, some researchers have conjugated Cur to poly(amidoamine) dendrimer (PAD) using hexachlorocyclotriphosphazene (HCCP) as a linker to form stable and homogeneous pH-responsive Cur-loaded nanospheres (HCCP-Cur-PAD, HCP NPs). *In vitro* experiments have shown that HCP NPs can enter the lysosomes through endocytosis. Thus, as a pH-responsive delivery system, HCP NPs can then rapidly dissociate and release Cur in the acidic environment of the lysosomes for the treatment of OP.^71^

### 3.2 Inorganic NPs

#### 3.2.1 Hydroxyapatite NPs

Hydroxyapatite NPs (HA NPs), as the primary inorganic component of bone, are highly similar to the minerals in the bone matrix. This similarity endows them with the capability to form direct chemical bonds with bone tissue and exhibit excellent biocompatibility^72^ and bone-targeting properties. Although pure HA has defects such as poor mechanical properties, this shortcoming can be significantly overcome through nanotechnology. HA NPs can serve directly as drug carriers or play a role in drug delivery after surface modification or structural modification. This tunability of structure and function makes HA NPs currently widely employed in fields such as bone tissue engineering.^73–75^

In the treatment of OP, HA NPs can be directly utilized as drug carriers to achieve precise regulation of bone metabolism by combining with anti-bone resorption drugs. For example, salmon calcitonin (SCT) is a drug used in the treatment of OP. It can specifically target and inhibit the activity of osteoclasts.^76^ By loading SCT onto HA NPs, a stable drug formulation of SCT-HA-NPs was prepared. The composite was shown to have enhanced mucosal permeability, subsequently leading to a significant increase in the targeted accumulation of SCT within bone tissue, ensuring a sustained effective local concentration of the drug in the skeletal area, which in turn inhibits osteoclast activity and ultimately increases BMD.^77^ In addition, recent studies have immobilized chitosan-nano-hydroxyapatite-alendronate composite microspheres onto the surface of polyetheretherketone, achieving dual regulation of osteogenesis and osteoclast inhibition, and significantly enhancing the osseointegration properties of the implant.^78^ Similarly, for targeted delivery of zoledronic acid, an anti-OP drug, researchers have developed drug-loaded HA NPs that demonstrate biphasic release properties. These NPs can enhance the enrichment of the drug within bone tissue by altering the drug release kinetics, thereby more effectively restoring the balance of bone metabolism.^79^ For bone-targeted delivery of anabolic drugs such as parathyroid hormone (PTH), the challenge lies in maintaining the drug activity while controlling its local side effects. PTH(1–34) with a negative charge can adsorb to HA nanorods (nHA-PTH(1–34)), which demonstrate an enhanced affinity for bone due to the high local concentration of calcium ions (Ca^2+^). Additionally, these nanorods are capable of solubilizing at pH 6.8, which is optimal for osteoclast activity, thereby facilitating the specific release of the hormone.^80^

In addition, doping ions or other bioactive molecules can endow HA NPs with additional regulatory functions. Researchers added zinc to HA NPs and developed risedronate/zinc-HA NPs in combination with risedronate. Integration of zinc into these particles was shown to increase the efficacy of the particles in enhancing the mechanical strength of the middle part of the femoral shaft and to improve outcomes in correcting the increased levels of urinary Ca and creatinine. In the dual-doped zinc–gallium HA NPs, the synergistic effect of zinc further enhanced the proliferation and differentiation of osteoblasts.^81^ This zinc modification was also illustrated to better maintain the microarchitecture of the bone cortex and trabeculae, thereby providing a more effective treatment for OP.^82^

Taken together, HA NPs have tunable physicochemical properties, a superior bone targeting ability, and significant drug-loading capacity. These attributes have collectively enabled the establishment of a comprehensive therapeutic platform that fuses bone-targeting drug delivery, metabolic regulation, and mechanical enhancement. The synergistic innovation strategy of ion doping and drug loading paves a new technological pathway for the precise treatment of OP and other bone metabolic diseases.

#### 3.2.2 Quantum dots

Quantum dots (QDs), as nanoscale inorganic semiconductor particles, not only hold unique fluorescence advantages in bioimaging and diagnostics but also serve as vectors for drug delivery.^83^ They have demonstrated a multidimensional application potential in the biomedical field.

Copper oxide QDs (CuO QDs), synthesized *via* mediation by *Angelica* species, are used to modify chitosan (CS), resulting in the formation of a CuO QDs/CS complex. This complex can significantly enhance osteoblast activity through interaction with the functional groups and potential compounds that bind to *Angelica*.^84^ In another study, researchers, combining graphene oxide QD (GOQD) with layered double hydroxide (LDH), constructed a composite nanocoating (GOQD/LDH) on the surface of magnesium (Mg) alloys. Experimental evidence confirmed that this coating improved the localized microenvironment for osteogenesis and rescued osteoblast apoptosis by activating mitochondrial autophagy to eliminate dysfunctional mitochondria. In a rat model, the coating improved the effects of bone regeneration and osseointegration, showcasing a promising design strategy for Mg alloy implants in the treatment of osteoporotic bone defects.^85^ Additionally, it expanded the therapeutic dimensions of bone tissue engineering through the innovative integration of QD with biomaterials.

#### 3.2.3 Magnetic NPs

Owing to their high specific surface area, quantum-size effects, and controllable magnetism, magnetic nanomaterials (such as those containing iron, nickel, cobalt, or their metal oxides) have great potential for a wide range of applications in fields like catalysis, energy storage, and biomedicine.^86–88^ Not only can they serve as Magnetic Resonance Imaging (MRI) contrast agents,^89^ but they can also function as vectors for drug delivery to augment the efficiency of diagnosis and treatment.^90–94^ These characteristics establish the groundwork for the precision treatment of OP using magnetic NPs (MNPs).

MNPs can play a therapeutic role by modulating bone cell activity. For example, iron oxide nanoparticles (IONPs) inhibit osteoclastogenesis by modulating the TRAF6-Sequestosome 1-Cylindromatosis signaling complex, which can be considered an alternative therapy for OP.^95^ Similarly, Bis-functionalized Bis/dextran/Fe_3_O_4_ NPs are specifically phagocytosed by osteoclasts, which can lead to the lysis of osteoclasts as a result of the increased temperature induced by the radiofrequency system. As revealed by *in vitro* experiments, this NP can significantly decrease the survival rate of osteoclasts. Additionally, these NPs possess MRI contrast capabilities, achieving the integration of diagnosis and treatment, and thus, these NPs hold certain potential in the treatment of OP.^96^

In addition to the direct killing of osteoclasts, MNPs can also deliver bioactive molecules. Gold-coated MNPs, serving as efficient carriers, are capable of loading bone marrow mesenchymal stem cell-extracellular vesicles. These vesicles deliver miR-150-5p to osteoblasts, thereby enhancing the proliferation and maturation of osteoblasts through the inhibition of matrix metalloproteinase 14 and activation of the Wnt/β-catenin pathway. This mechanism is particularly applicable to the promotion of bone metabolism in diabetic OP, offering a potential novel drug delivery method.^97^

#### 3.2.4 Gold NPs

Gold NPs are the most stable metal NPs. With their excellent biocompatibility and low toxicity, they exhibit broad application prospects in the field of biomedicine.^98,99^ Gold NPs have found extensive application in bone regeneration. For example, silver (Ag)-gold-HA NPs, prepared by incorporating Ag and gold NPs into HA NPs, can significantly promote bone regeneration and fracture healing.^100^ In addition to directly participating in bone tissue reconstruction, gold NPs can also be combined with polymer materials to form functional scaffolds to enhance bone regeneration. Fabricate 3D scaffold based on poly (l-lactic acid)/polycaprolactone (PCL) matrix polymer containing gelatin nanofibers and gold NPs stimulate osteoblast proliferation at a concentration of 80 ppm gold NPs. It also guides new bone formation through the three-dimensional structure of the scaffolds.^101^

In the treatment of OP, gold NPs can inhibit the receptors for Receptor Activator of Nuclear Factor κ B Ligand (RANKL) and macrophage colony-stimulating factor (M-CSF). This inhibition significantly reduces the bone resorption activity of osteoclasts, endowing gold NPs with potential as a therapeutic agent for disorders (such as OP) related to bone metabolism *via* altered number and activity of osteoclasts.^102^ Apart from directly regulating cell behavior, gold NPs can also serve as highly efficient drug carriers. They can deliver small molecule drugs, such as Ångstrom-scale gold particles by surface conjugation with alendronate, achieving dual functions of osteoblast activation and osteoclast inhibition. In a mouse OP model, alendronate-loaded Ångstrom-scale gold particles maintained bone microarchitecture and strength, prevented bone resorption, and improved BMD, demonstrating potential applicability in the treatment of OP.^103^ Gold NPs modified with β-cyclodextrin (β-CD) were used to load Cur, preparing GNPs covered with CD/Cur inclusion complex (Cur-CGNPs). This complex inhibited the formation of multinucleated osteoclasts in RANKL-induced bone marrow-derived macrophages (BMMs) without exhibiting cytotoxicity, and in the mouse OP model, Cur-CGNPs significantly enhanced BMD and prevented bone resorption, suggesting their potential as an effective therapeutic agent for the prevention and treatment of OP.^104^

Polyethylene glycol-modified hollow GNPs, upon loading with α-lipoic acid, are capable of effectively scavenging intracellular excessive ROS. This action suppresses the level of cellular oxidative stress, thereby substantially enhancing the viability of osteoblasts. In addition, in cellular experiments, these GNPs were able to increase gene expression of Bone Morphogenetic Protein (BMP) −2, Runt-related transcription factor 2 (Runx2), and osteocalcin (OCN) and enhance osteoblast differentiation, holding potential therapeutic value in the treatment of OP.^105^

Gold NPs have been shown to play a role in the realm of gene therapy for OP. As previously discussed, the overexpression of the *SOST* gene leads to OP. The encapsulation of PEI and SOST siRNA onto CS-modified Gold NPs results in the formation of PEI/siRNA/CS-Gold NPs. This process has been shown to enhance the stability and transfection efficiency of siRNA, overcoming its short half-life and low payload issues. Treatment with this gene therapy was shown to significantly downregulate expression of the *SOST* gene by threefold and at the same time to upregulate the expression of osteogenic markers Runx2 and alkaline phosphatase (ALP), promoting bone formation. In addition, this nanotherapeutic treatment was shown to be safe.^106^

In addition to Gold NPs, other metal and metal oxide NPs also hold potential in the treatment of OP. Calcein-functionalized Ca–aluminum-LDH (CALC) nanosheets enhanced the acid-base balance of the bone microenvironment through acid neutralization, thereby inhibiting osteoclast activity.^107^ Concurrently, the release of Ca^2+^ from these nanosheets led to the formation of Ca phosphate NPs by promoting the activation of alternatively activated macrophages and Treg cells *via* immune modulation, which reshaped the immune microenvironment.^108^ In the mouse model of OP, CALC nanosheets significantly elevated the femoral bone volume fraction from 6.2 to 10.7, effectively reversing the pathological progression of OP.^109^ Similarly, the multifunctional nanoplatforms, composed of Ca-based upconverting NPs and Mg–organic frameworks, acquire bone targeting and pH responsiveness. In the acidic microenvironment of OP, nanoplatforms gradually degrade, causing the release of Mg^2+^ and Ca^2+^, which synergistically complement the direct involvement in the process of bone mineralization and promote the formation of HA.^110^

#### 3.2.5 Bioactive glass NPs

Bioactive glass is a silicate-based amorphous material developed in 1969 by Larry Hench and others. The core mechanism of its application in bone tissue involves promoting the regeneration of bone *via* surface apatite crystallization and the release of ions (such as Ca^2+^ and silicon ions).^111^ In addition, mesoporous bioactive glass NPs (MBG NPs) can efficiently load biomolecules or drugs due to their high specific surface area and ordered mesoporous structure, offering novel material option for the treatment of bone-related diseases.^112–115^

In the treatment of OP, researchers have enhanced and expanded the functionality of MBG NPs through strategies such as ion doping, drug loading, and the design of composite scaffolds. For example, strontium (Sr)-substituted MBG NPs (Sr-MBGNPs) and icariin were co-embedded within 3D-printed PCL scaffolds.^116^ The optimized concentration of Sr-MBGNPs within these scaffolds ensured sustained ionic release, exceptional hydrophilicity, and bioactivity while maintaining scaffold integrity. These scaffolds demonstrated dual functions: enhancing osteoblast proliferation and differentiation and inhibiting osteoclasts, thus providing a localized delivery platform for osteoporotic bone defects.^116^ Similarly, β-CD-modified MBG NPs (CD-MBGNPs) were integrated with silk fibroin (SF) loaded with exogenous estradiol (E2) to form a composite material (E2@CD-MBGNPs/SF). This composite utilized the mesoporous structure of MBG, the high affinity of β-CD for hydrophobic drugs, and the biocompatibility of SF to facilitate a sustained release of E2. The released E2 inhibits the formation of multinucleated osteoclasts and osteoclast activity, concurrently reducing levels of bone resorption-related markers, such as tartrate-resistant acid phosphatase. This material is not only expected to serve as a minimally invasive filler for osteoporotic bone defects but also offers insights into the treatment of OP.^117^ In addition, following the composite formation of boron-containing MBG (B-MBG) with the novel *p*(*N*-isopropylacrylamide-*co*-butyl methacrylate) (PIB) nanogel, the incorporation of B-MBG improved the mechanical strength of the PIB nanogels. It also enhanced its bone regeneration capability, making it suitable for repairing bone defects with irregular shapes.^118^ This could also potentially open a new avenue for the treatment of OP.

#### 3.2.6 Silica NPs

In recent years, due to their unique advantages such as excellent chemical stability and biocompatibility,^119^ silica NPs have gained widespread application in various fields, including drug delivery, bioimaging, and biosensors.^120^ Their structures predominantly encompass two types: mesoporous (MSNs) and core/shell. Among these, MSNs hold exceptional drug-loading capabilities owing to their high specific surface areas and porous outer shells, thereby playing a significant role in the treatment of OP.^121^

Previous studies have verified that bioactive silica NPs (50 nm spherical silica core/shell structured NPs) can stimulate the differentiation and mineralization of osteoblasts, inhibit the differentiation of osteoclasts, and significantly increase the BMD *in vivo*.^122^ However, the efficacy of the NPs was limited by their lack of antioxidant properties and low levels of cell adhesion properties. To address this deficiency, researchers have developed thiolated MSNs (MSN–SH), which can reduce cellular damage by neutralizing ROS within cells.^123^ It was verified that these NPs can enhance the proliferation of osteogenic progenitor cells in mice. More notably, MSN–SH can induce bone formation without relying on any osteogenic supplements. This was achieved by upregulating the expression of osteogenic gene markers such as Runx2, ALP, OCN, and osteopontin. Consequently, it showed potential as a complementary and alternative therapy for OP.^123^

In addition to their direct function after activation, MSNs, following surface modification, can also serve as highly efficient drug delivery vehicles, contributing to the treatment of OP. After the surface of MSNs is modified with the cationic polymer PEI, these NPs can enhance the cell viability of pre-osteoblasts and human mesenchymal stem cells (MSCs) by loading pleiotrophin. Additionally, they increase the gene expression of ALP and Runx2. *In vitro* experiments have demonstrated that this nanometer system can exert potent osteogenic differentiation effects without the need for a culture medium that promotes osteogenic differentiation. This breakthrough overcomes the limitations of traditional bone induction-dependent mediums, thereby offering a novel approach for the treatment of OP.^124^

Doping with metal ions can also further optimize the bone regeneration function of MSNs. Using Ca, gallium (Ga), or a combination of these elements as dopants, it was observed that the incorporation of Ca^2+^ resulted in a reduction of the specific surface area of the NPs by approximately 50%. Conversely, Ga doping maintained the stability of the material's physicochemical properties.^125^ In cellular experiments, Ga-containing MSNs not only enhanced osteogenic activity but also inhibited the osteoclastic differentiation of macrophages. This positioned them as promising candidates for applications in bone tissue regeneration, especially in non-physiological or pathological conditions such as OP.^125^

**Fig. 2:**
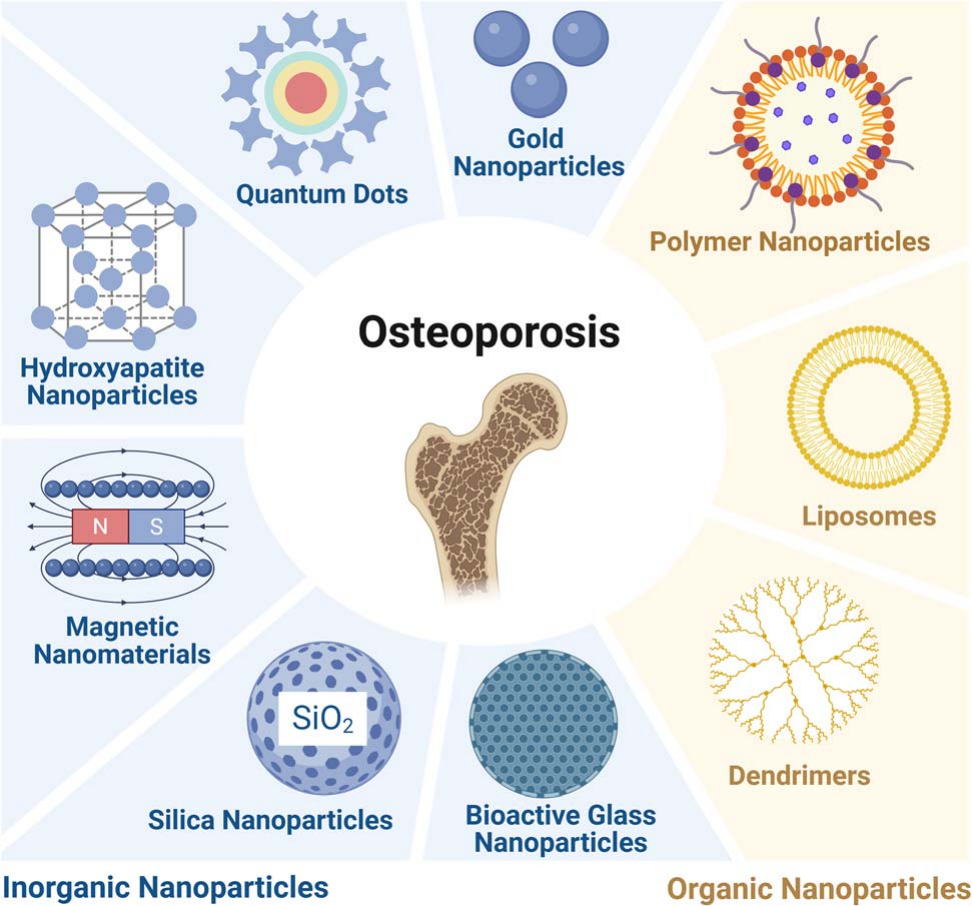
Different types of nanoparticles tested and applied in the treatment of osteoporosis. Organic nanoparticles include polymeric nanoparticles, liposomes, and dendrimers, and inorganic nanoparticles include quantum dots, gold nanoparticles, hydroxyapatite nanoparticles, magnetic nanoparticles, silica nanoparticles, and bioactive glass nanoparticles. Created in https://BioRender.com.

Taken together, both the organic and inorganic NPs discussed above (as summarized in Fig. 2) have demonstrated multi-level and multi-target modulation and functions in the treatment of OP. As nanomedicine technology progresses, these materials are anticipated to transition from laboratory research to clinical applications, offering safer and more effective treatment modalities for OP patients.

## 4. Mechanisms of action of NPs in treating OP

**Fig. 3:**
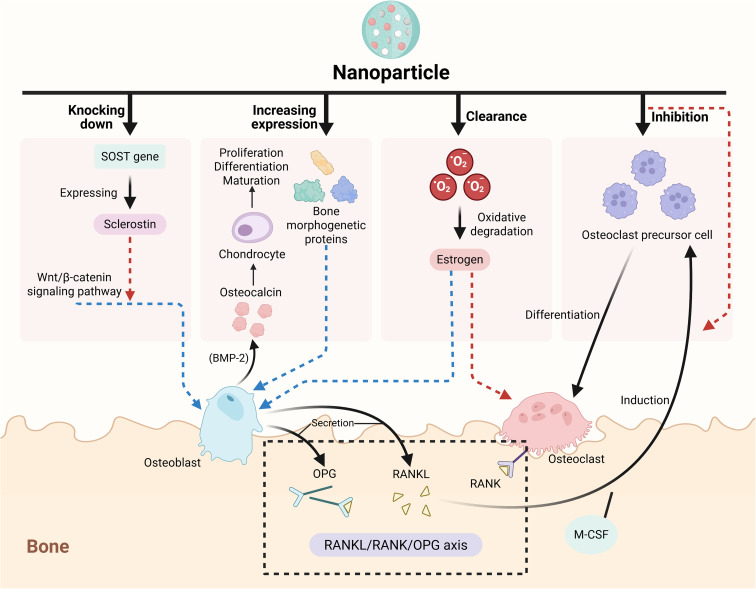
Related action mechanisms of nanoparticles in the treatment of osteoporosis. In the treatment of osteoporosis, nanoparticles can modulate some key relevant signaling pathways and genes that are important in regulating bone metabolism (with blue arrow indicating promotion and red ones indicating inhibition). Nanoparticles can specifically knock down the *SOST* gene, reduce the production and secretion of sclerostin, relieving the inhibition of the Wnt pathway and promoting the differentiation of osteoblasts and bone formation. Nanoparticles can also promote the expression of bone morphogenetic proteins, thereby facilitating the expression of osteogenesis-related genes and promoting the osteogenesis process. In addition, nanoparticles can inhibit the differentiation of pre-osteoclasts and reduce the maturation of osteoclasts and their bone resorption function by interfering with the RANKL/RANK/OPG axis. Nanoparticles can also scavenge ROS in the body, reduce the oxidative degradation of estrogen, and inhibit the formation of osteoclasts. Moreover, nanoparticles can also directly target and regulate pre-osteoclasts, thereby inhibiting the maturation of osteoclasts and the bone resorption process. Created in https://BioRender.com.

After NPs overcome traditional delivery limitations and offer new directions for osteoporosis (OP) treatment, the key to understanding their efficacy is analyzing their mechanisms of action—different NPs vary in bone metabolism regulation pathways but all relate to promoting bone formation and inhibiting bone resorption. The following summarizes these core mechanisms and explains how NPs restore bone metabolic balance (Fig. 3).

### 4.1 Targeted regulation of key cells involved in bone metabolism

The maintenance of bone homeostasis depends on the dynamic balance of osteoblasts and osteoclasts. In patients with OP, there is often a significant increase in the number and activity of osteoclasts, leading to bone loss due to the release of acids and proteases that degrade the bone matrix.^126^ On the other hand, the differentiation of osteoblasts is inhibited or their function diminished, which further exacerbates the imbalance in bone metabolism. As osteogenic precursor cells, MSCs have the critical ability to differentiate into osteoblasts, which is essential for bone formation and regeneration. However, under conditions of aging or pathology, these cells tend to differentiate into adipocytes (fat-forming cells) rather than osteoblasts, thereby further decreasing bone formation.^127,128^

**Fig. 4:**
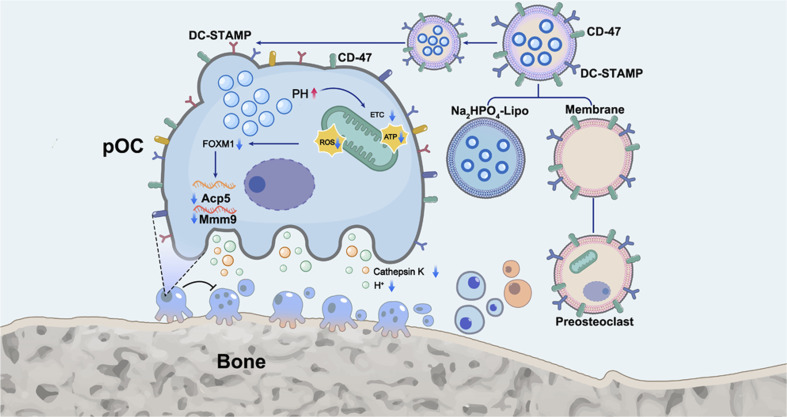
The construction of Na_2_HPO_4_@Lipo-pOCm nanovesicles and their spatiotemporal selective delivery mechanism in pre-osteoclasts. Membranes extracted from pre-osteoclasts (pOCm) fuse with liposomes loaded with Na_2_HPO_4_ (Lipo) to form a composite nanovesicle. After membrane fusion, this nanovesicle enters pre-osteoclasts and releases Na_2_HPO_4_, which neutralizes intracellular H^+^, thereby reducing mitochondrial membrane potential and suppressing ROS production. In addition, it markedly down-regulates the transcription factor FOXM1 and its downstream bone-resorbing genes (such as Acp5, CTSK, *etc.*). This cascade inhibits osteoclast maturation and bone-resorbing activity, achieving therapeutic effects against osteoporosis. Source: this publication is licensed under CC-BY-NC-ND 4.0. Copyright © 2023 The Authors. Published by American Chemical Society.^129^

Restoring bone homeostasis is essential for the treatment of OP, and NPs can effectively target and modulate the functions of these three types of cells to exert therapeutic effects. For example, Na_2_HPO_4_@Lipo-pOCm NPs (a novel nanoparticle encapsulating Na_2_HPO_4_ in liposomes hybridized to preosteoclast membranes) can inhibit osteoclast maturation and bone resorption activity by targeting preosteoclasts and membrane fusion to release Na_2_HPO_4_ into preosteoclasts. They can reduce intracellular H^+^ concentration, thereby inhibiting osteoclast formation and bone resorption activity, while upregulating the osteogenic potential of MSCs^129^ (Fig. 4). Iron oxide NPs promote the differentiation of osteoblasts by stimulating the activation of the Wnt signaling pathway. Furthermore, they can also inhibit the formation of osteoclasts by modulating the Transforming Growth Factor Beta 6-P2Y12 receptor signaling complex to treat OP.^130,131^

### 4.2 Effects on regulating relevant genes and signaling pathways

At the molecular signaling pathway level, NPs can regulate or interfere with key regulatory nodes such as the RANKL/RANK/osteoprotegerin (OPG) axis (the key signal pathway for promoting osteoclastogenesis) and the BMP/Smad and Wnt/β-catenin signaling pathways (the key signals for promoting osteoblast differentiation and osteogenesis). The RANKL/RANK/OPG axis is the core pathway that regulates osteoclast differentiation. Upon binding to its receptor RANK, RANKL activates signaling pathways such as NF-κB and MAPK, which promote osteoclast differentiation; whereas OPG, serving as a decoy receptor for RANKL, can inhibit this process. Both RANKL and OPG are secreted by osteoblasts, and the balance between them significantly regulates the bone resorption activity of osteoclasts. A higher ratio of RANKL to OPG results in increased bone resorption activity, whereas a lower ratio leads to decreased bone resorption activity.^132,133^ Gold NPs significantly block the fusion process of osteoclast precursor cells induced by the combination of RANKL and M-CSF. They also effectively limit the migratory capability of osteoclasts and inhibit the formation of actin ring structures, ultimately decreasing the bone resorption function of osteoclasts.^102^

BMPs are a group of versatile growth factors that play a crucial role in the development of bone tissue and the maintenance of metabolic balance. Among these, BMP-2, 4, 5, 6, and BMP-7 demonstrate the greatest capacity to promote bone formation. For example, BMP-2 enhances the differentiation of osteoblasts and expression of bone protein OCN and it also stimulates the proliferation and maturation of chondrocytes, which plays an important role in the process of endochondral ossification.^134^ Additionally, BMP-7 increases ALP activity and the deposition of extracellular calcium Ca, thereby facilitating the differentiation of osteoblasts.^135^ While BMPs mostly promote osteogenesis-related gene expression through activation of Smads proteins, there are also non-classical Smad-independent BMP signaling pathways (such as TGF-β activation kinase1, MAPKs and so on)^136^ which can precisely modulate the osteogenesis process. Selenium NPs can significantly increase BMP-2 expression in cells, leading to the phosphorylation of downstream MAPKs, facilitating the nuclear translocation of β-catenin, and promoting the expression of genes associated with osteogenesis.^137^

The hardened protein called sclerostin, encoded by the *SOST* gene, functions as an inhibitor of the Wnt/β-catenin signaling pathway. Excessive expression of sclerostin impedes the differentiation of osteoblasts and the formation of bone. In contrast, delivery of SOST siRNA *via* MSNs specifically knocked down the *SOST* gene and deregulated the Wnt pathway, thereby activating the expression of osteoblast differentiation-related genes (such as Runx2, Osterix) and promoting the secretion of osteogenic markers (ALP and OCN).^138^

Moreover, in addition to the aforementioned pathways, signaling nodes such as Notch and phosphatidylinositol 3-kinase/protein kinase B are also involved in the regulation of bone metabolism.^139,140^ Through precision modulation of these signaling pathway nodes, nanotechnology achieves multi-dimensional intervention in the dynamic balance of osteogenesis and osteoclast genesis. The advancement of nanotechnology is developing a systematic solution for the treatment of OP that spans from gene silencing to activation or inactivation of key signaling pathways involved in bone metabolism.

### 4.3 Effects on other key factors, hormones, and the microenvironment

**Fig. 5:**
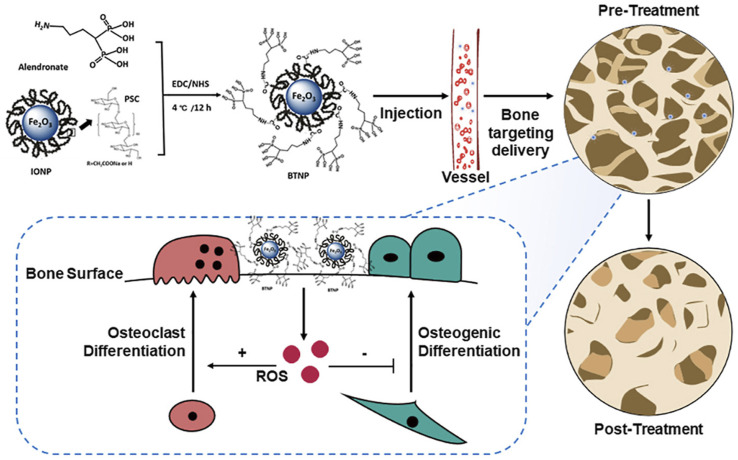
The preparation, delivery, and mechanism of action of bone-targeted antioxidant nano-iron oxide (BTNP) for treating postmenopausal osteoporosis. Iron oxide nanoparticles (IONP), polymer/phosphate ligand (PSC), and alendronate are combined under EDC/NHS activation at 4 °C for 12 h to synthesize bone-targeted nanoparticles (BTNP), which are then injected intravenously for targeted delivery to bone tissue. The figure compares bone microarchitecture before and after treatment. Below the image, it is further described that BTNP can modulate the generation of reactive oxygen species (ROS), manifested as promotion of osteoclast differentiation (+) and inhibition of osteoblast differentiation (−), thereby regulating bone metabolism. Source: this figure is reproduced from Liming Zheng *et al.*^142^ Copyright 2021, reproduced with permission from Elsevier.

The development of OP is also closely related to the local acidified microenvironment, inflammation (with elevated levels of pro-inflammatory factors such as cytokines TNF-α and IL-6), and hormonal imbalances (such as estrogen deficiency). While estrogen can treat OP through a dual mechanism of inhibiting the bone resorption activity of osteoclasts and promoting the proliferation and differentiation of osteoblasts,^141^ its clinical application is constrained by limitations in metabolic stability, and ROS in the body can accelerate the oxidative degradation of estrogen, reducing its bioavailability. To address this issue, IONPs can act as scavengers of ROS to treat OP by protecting estrogen from degradation^142^ (Fig. 5). Similarly, through modulation of intracellular ROS levels, cerium oxide NPs reduce osteoclast number by inducing apoptosis of their precursor cells, BMMs, although cerium oxide NPs can promote osteoclast formation at lower concentrations.^143^

Taken together, in the treatment of OP, NPs demonstrate synergistic effects and functions in modulating key signaling pathways, targets and factors, resulting in inhibition of osteoclast genesis, promotion of osteogenesis, and regulation of the bone microenvironment.

## 5. Advantages of NPs and future work needed

Owing to their unique size effects, high specific surface area and reactivity, and superior biocompatibility, as well as their abilities in targeted delivery of drugs and in modulating key signal pathways important for regulating bone metabolism, various types of NPs (including both organic and inorganic NPs) have been tested and applied for R&D for OP treatment.

NP-based therapeutics represent a groundbreaking strategy in OP treatment with some obvious advantages. By designing bone-targeted nanocarriers (such as MSNs modified with Bis or polyethylene glycol), the precise delivery of therapeutic molecules (such as siRNA and osteogenesis inhibitors) can be achieved. By combining synergistic effects of gene regulation and bioactive molecules, osteoblast differentiation can be promoted, and osteoclast differentiation and activity can be inhibited. In addition, by enhancing targeted delivery and drug release, drugs can be protected from degradation, improving their stability and bioavailability. Targeted delivery with NPs also makes it possible to load the drug with lower doses that may be required, reducing the frequency of drug use, the risk of systemic side effects and/or potential toxicity associated with traditional oral medications.^144^ Furthermore, NPs are highly customizable, allowing for modifications in size and surface functional groups. This characteristic enables them to be adaptable to the needs of personalized treatment and may improve the treatment outcomes under specific conditions.

Despite these advantages, the field also faces some significant challenges that hinder the clinical translation and industrial production of NPs, including the lack of intra-batch size uniformity, batch-to-batch reproducibility, *in vivo* stability, and long-term storage stability of the current artificially prepared NPs.^145,146^ Long-term toxicity data are still lacking, and most of the innovative nano-delivery systems have been evaluated through preclinical investigations and are still in the preliminary/early research stage.^35^ These challenges are the central focus of current research in the field and represent the critical barriers that must be overcome to translate findings from the lab to the clinic.

Future research is required for further optimization of their safety of metabolism *in vivo*, their long-term biological effects, and scaling of their preparation processes, as well as development of new nanometer systems with synergistic multi-mechanisms, which can facilitate their translation into clinical applications. Future research should focus on developing intelligent, stimuli-responsive nanosystems (such as pH- or enzyme-triggered drug release) and promoting their advancement through standardized preclinical and clinical studies that adhere to rigorous drug evaluation standards. With the breakthroughs in the surface functionalization technology of nanomaterials and the advancement of clinical trials, this field is expected to achieve a leap from experimental research to clinical treatment.

## 6. Conclusion

NPs represent a revolutionary solution for overcoming the limitations of traditional OP drugs, leveraging their unique size effects, engineerable surface properties, excellent biocompatibility, and diverse carrier forms. This review has systematically elaborated on the significant potential demonstrated by various types of NPs in OP treatment, achieved through precise bone targeting, controlled drug release, and multi-pathway synergistic regulation of bone metabolism. Despite the significant promise shown in preclinical research, the clinical translation of NPs faces key challenges, including the standardization of preparation processes and the evaluation of long-term *in vivo* safety. Future research efforts should focus on developing “intelligent” nanosystems with multi-mechanism synergistic therapeutic capabilities, conducting in-depth assessments of their long-term biological effects, and advancing breakthroughs in scalable production technologies.

**Fig. 6:**
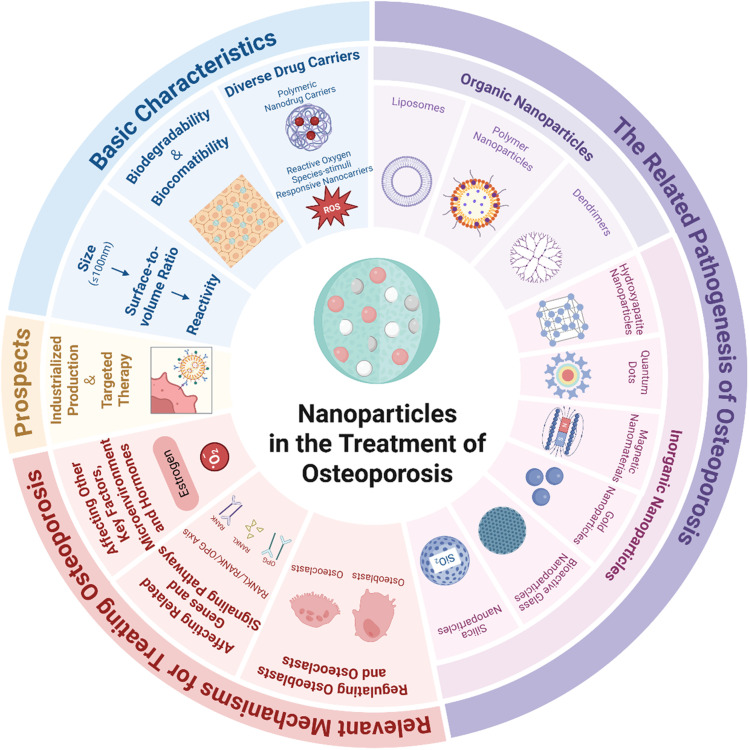
An overview of nanoparticles in the treatment of osteoporosis. It includes the basic properties of nanoparticles, different types of nanoparticles tested and/or applied in the research and development for the treatment of osteoporosis, mechanisms of action of NPs for treating osteoporosis, the application advantages and the prospects of NPs. Created in https://BioRender.com.

In summary, NP-based therapies constitute a highly promising new paradigm in the field of OP treatment. With the deep integration of nanotechnology and biomedicine, we have reason to anticipate that this strategy will open a new chapter in the precise treatment of osteoporosis (Fig. 6).

## Author contributions

Conceptualization, Y. L. and Z. L.; original draft preparation, Y. L., Z. L., J. L. and C. J. X.; data collection, W. W. and J. Z.; project administration, Y. Z.; validation, Y. L.; supervision, Y. L.; funding acquisition, Y. Z.; review and editing, Y. L., Y. Z. and C. J. X.; Y. L. and Z. L. contributed equally to this work.

## Conflicts of interest

There are no conflicts to declare.

## Data Availability

Data sharing is not applicable to this article as no new data were created or analyzed in this study.
